# Giant splenic arteriovenous shunt with arterial and venous aneurysmal dilatation causing portal hypertension and gastric varices

**DOI:** 10.1016/j.jvscit.2026.102538

**Published:** 2026-09-29

**Authors:** Muhammad Anza Talal Khan, Syed Saad Ali Chishti, Ubaid Ullah, Avinash Deshwal, Aine Stakelum, Kenneth Thornton, Elrasheid AH. Kheirelseid

**Affiliations:** Department of Vascular Surgery, Beaumont Hospital and RCSI, Dublin, Ireland

**Keywords:** Arteriovenous shunt, Splenic artery, Hypertension, Portal esophageal, Gastric varices, Splenectomy

## Abstract

A 59-year-old woman presented with recurrent hematemesis and generalized weakness. Imaging revealed a giant high-flow arteriovenous shunt between markedly dilated splenic arterial and venous systems, associated with an 8 × 5 cm splenic arterial aneurysmal component, portal hypertension, and gastric fundal varices. Angiography demonstrated high flow through markedly dilated vessels, precluding safe endovascular embolization. The patient underwent open splenectomy with complete excision of the vascular lesion, achieving full symptom resolution and complete resolution of gastric varices at 6-month follow-up. Giant splenic arteriovenous shunts are exceedingly rare, and no consensus on their optimal management has been established. Management should be individualized according to vascular anatomy and flow characteristics, with splenectomy reserved for lesions unsuitable for endovascular treatment. The etiology of the arteriovenous shunt remained uncertain, as neither a congenital origin nor a remote post-traumatic origin could be established.

Arteriovenous malformations (AVMs) are vascular malformations in which an artery communicates directly with a low-pressure vein through an abnormal network of arterioles, posing diagnostic and surgical challenges that vary with anatomical location, type, and hemodynamic characteristics. AVMs may be congenital, arising from aberrant vascular development, or acquired following trauma, hormonal influences, or other vascular insults.^1^, ^2^ Regardless of the etiology, they carry a significant risk of rupture and life-threatening hemorrhage.^2^ Although splenic artery aneurysms occur in 1% of the population, arteriovenous communication between the splenic artery and vein is rare, and no consensus on their optimal management has been established.^3^ We report a giant splenic arteriovenous shunt causing portal hypertension and gastric varices, highlighting the diagnostic and therapeutic challenges of this rare vascular lesion of uncertain etiology.

The patient has provided written consent for the publication of her case details and accompanying images.

## Case presentation

A 59-year-old woman was transferred to our vascular service from another hospital for management of a giant splenic arteriovenous shunt. She initially presented with two self-limiting episodes of hematemesis, generalized weakness, and a brief collapse, having reported preceding mild-abdominal discomfort and increased urinary frequency. She was hemodynamically stable on arrival. She was a nonsmoker without a history of excessive alcohol consumption, had undergone spinal surgery following a road traffic accident 30 years earlier with recalled abdominal wall bruising but no known internal injury; contemporaneous medical records and imaging from this remote admission were unavailable for review. Her sister had died of liver failure. She took only over-the-counter analgesics and esomeprazole regularly, with allergies to codeine and nitrofurantoin.

Initial investigations at the referring hospital included upper- and lower-gastrointestinal endoscopy and computed tomography (CT) imaging. Upper-gastrointestinal endoscopy, performed for hematemesis, melena, and anemia, demonstrated no intraluminal blood, esophageal or gastric varices; colonoscopy revealed a benign cecal polyp removed by snare polypectomy. Initial contrast-enhanced CT demonstrated marked gastric fundal varices and a large arteriovenous shunt with direct communication between the splenic arterial and venous systems, associated with an 8 × 5 cm splenic arterial aneurysmal component. On transfer, abdominal and systemic examinations were unremarkable, and hemoglobin was 11 g/dL. She was commenced on intravenous cephalexin, esomeprazole, paracetamol, and cyclizine. Interventional radiology was consulted. Catheter angiography demonstrated a very high-flow arteriovenous shunt, characterized by rapid arteriovenous shunting through direct communication between the markedly dilated splenic arterial and venous systems (Fig 1). Subsequent CT angiography confirmed persistent communication between the splenic arterial and venous systems at the splenic hilum, with dilatation of the portal venous system and prominent gastric fundal varices (Fig 2). No active hemorrhage was seen, though hemoglobin fell to 8.3 g/dL over the following days, accompanied by a further episode of hematemesis and melena with stool positive for occult blood; she received 1 unit of packed red blood cells.

**Fig 1:**
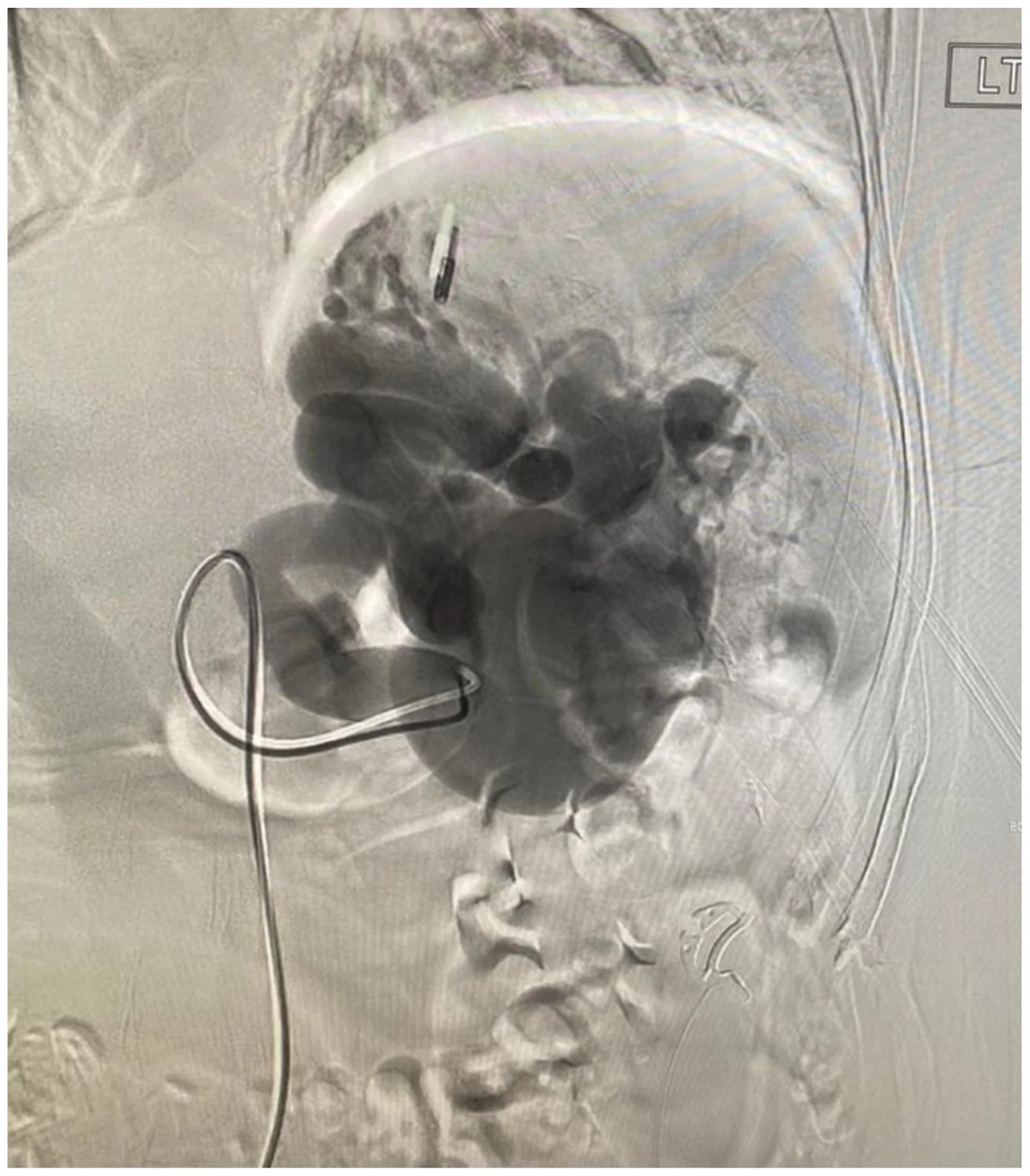
Angiogram of the splenic arteriovenous malformation (AVM), demonstrating high-flow communication between the splenic artery and vein with marked dilatation of both vessels.

**Fig 2:**
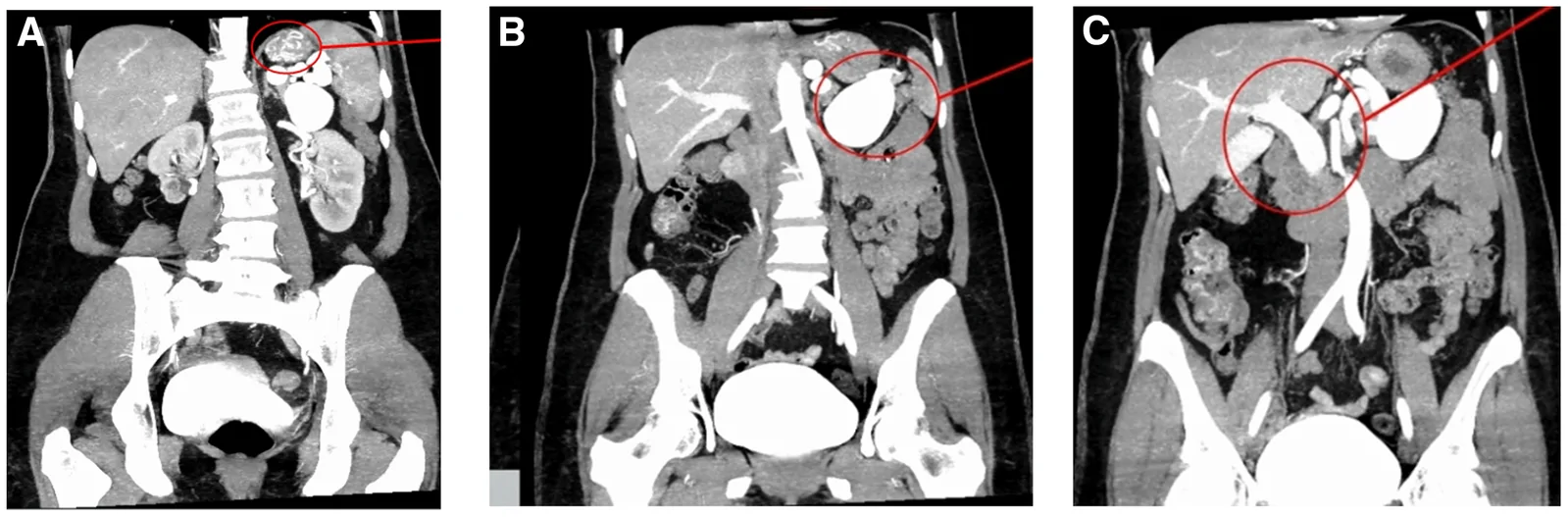
**A,** Computed tomography (CT) scan showing gastric fundal varices (*encircled*). **B,** CT scan showing the enlarged splenic arteriovenous malformation, measuring 8 × 5 cm (*encircled*). **C,** CT scan showing portal venous reflux of contrast (*encircled*).

Given ongoing bleeding and risk of catastrophic hemorrhage from portal hypertension, splenectomy with excision of the vascular lesion was planned. Preoperative liver function tests, international normalized ratio, and hemoglobin (9.7 g/dL) were normal. Via midline laparotomy extended to the left-upper quadrant, the splenic artery and vein were dissected and controlled to isolate the arteriovenous shunt (Fig 3), with marked dilatation of the associated arterial and venous structures. The spleen was mobilized, and splenectomy with excision of the vascular lesion completed (Fig 4). An inadvertent diaphragmatic injury was promptly repaired, and chest and abdominal drains placed. Histopathology demonstrated vascular abnormalities with atheromatous change and fibrin thrombus, without malignancy.

**Fig 3:**
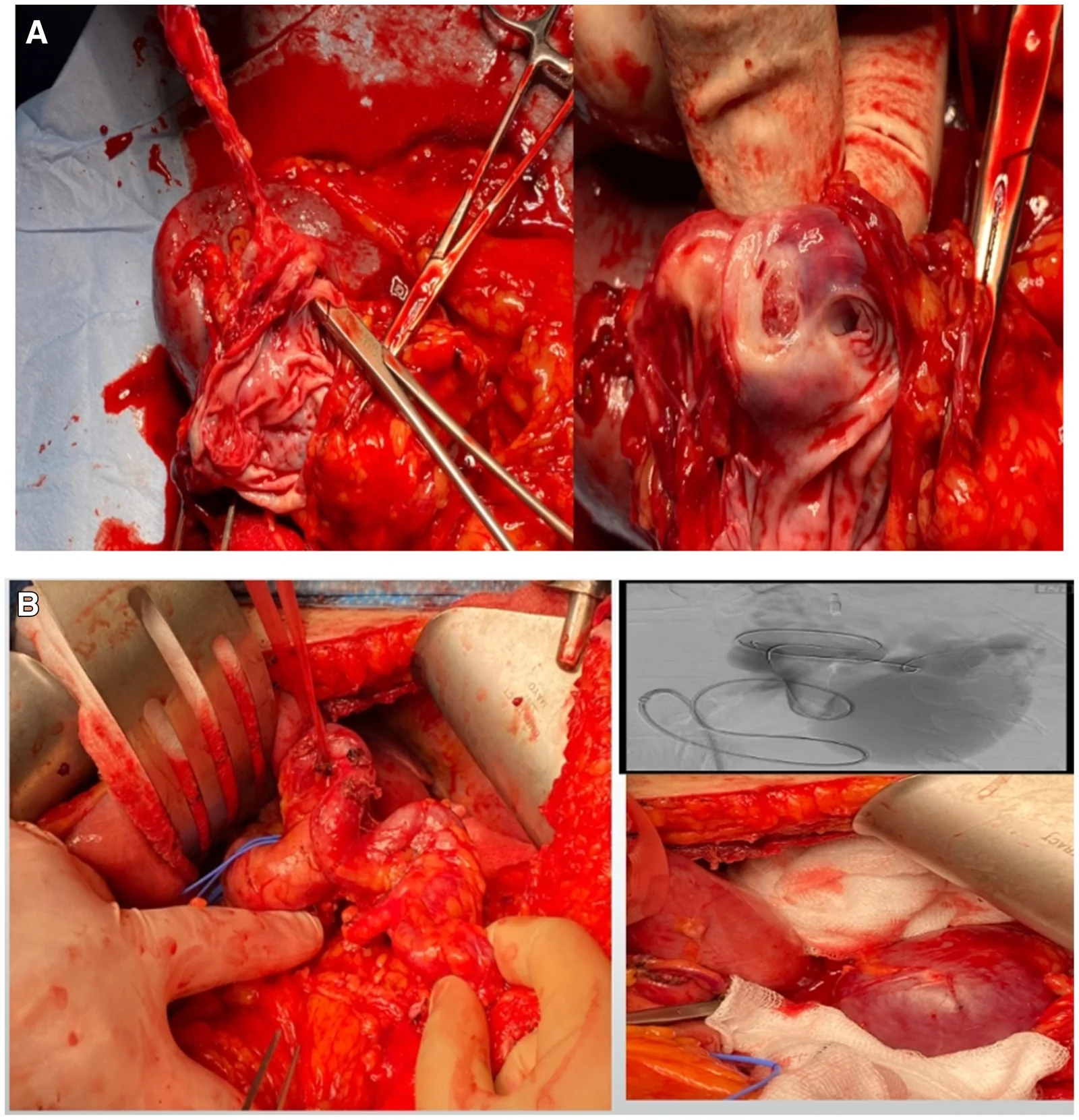
**A,** Intraoperative image of the spleen, splenic vasculature, and arteriovenous malformation. **B,** Intraoperative image of the spleen, splenic vasculature, and arteriovenous malformation, with corresponding angiography imaging.

**Fig 4:**
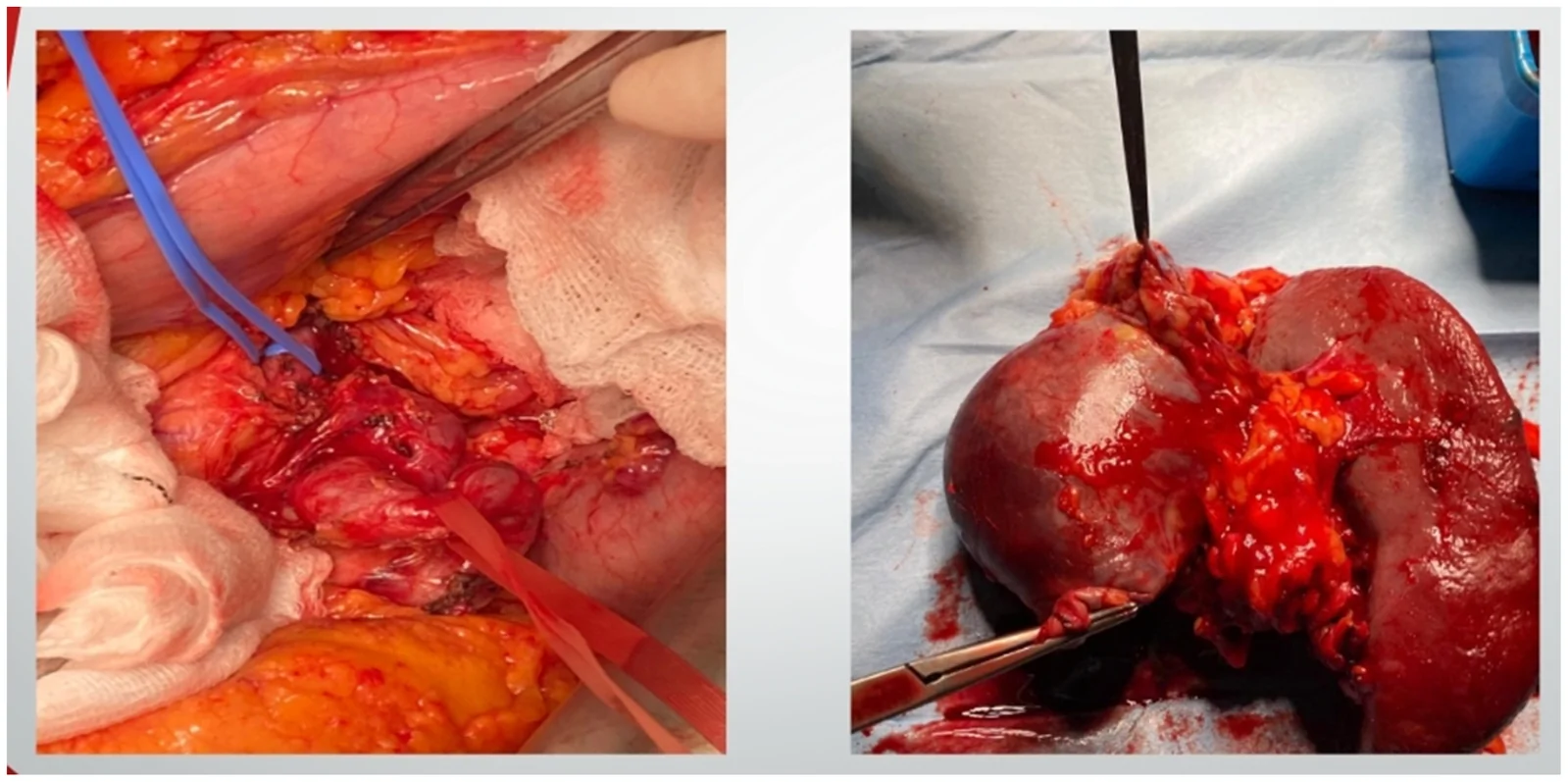
Excised splenic artery and vein with the associated arteriovenous malformation, measuring 8 × 5 cm.

Postoperatively she developed left-sided atelectasis and a small apical pneumothorax, and required a further transfusion. Drain fluid amylase was normal, excluding pancreatic injury; C-reactive protein peaked at 13.9 mg/L and normalized before discharge. She was commenced on lifelong penicillin V prophylaxis and referred for postsplenectomy vaccination. Total parenteral nutrition was used until postoperative day 3, followed by oral intake. Persistent fever prompted repeat imaging showing only minor postoperative changes with trace pelvic/subphrenic fluid and no leak or hemorrhage; atelectasis improved with intensified chest physiotherapy. She was discharged hemodynamically stable and afebrile, and remained well at 6-month follow-up with no recurrent bleeding and complete endoscopic resolution of gastric varices.

## Discussion

The etiology of this patient’s giant splenic arteriovenous shunt remains uncertain. A congenital origin could not be established, as dedicated whole body or cerebral vascular imaging for additional vascular malformations and genetic testing for an inherited vascular disorder were not performed. Conversely, the remote history of abdominal trauma raises the possibility of an acquired post-traumatic arteriovenous communication; however, contemporaneous records and imaging were unavailable to confirm splenic injury. Therefore, neither a congenital nor an acquired origin can be definitively established. Hamidi et al^4^ found that initial CT after blunt abdominal trauma is generally sufficient for diagnosis, with follow-up scans rarely identifying new findings, though the spleen remains the most commonly injured organ; based on our experience, routine follow-up imaging is not warranted when the initial scan is normal, though a follow-up CT at approximately 1 year may be prudent when solid organ injury has been managed conservatively. Iannaccone et al^5^ described a patient with only mild symptoms and an unremarkable ultrasound who collapsed and died, with autopsy revealing a splenic AVM, underscoring that these lesions may remain clinically silent before presenting catastrophically.

Giant splenic arteriovenous communications associated with aneurysmal dilatation are rarely reported. Selected relevant reports illustrate the variability in their presentation and management. Agrawal et al^6^ described a 10 cm splenic artery aneurysm with hilar AVM managed by splenectomy and distal pancreatectomy. Ibrahim et al^7^ reported a giant venous aneurysm with AVM treated successfully by endovascular balloon occlusion, whereas Urbano et al^8^ treated an 8 cm atypical splenic AVM with transarterial embolization using ethylene-vinyl alcohol copolymer. Two principal treatment modalities exist: endovascular embolization/coiling and open surgical excision with splenectomy. In our patient, endovascular management was attempted but deemed unsafe because of high flow and vessel dilatation, necessitating open splenectomy with excision of the vascular lesion. In very high-flow shunts, rapid transit across the arteriovenous communication may result in uncontrolled migration of embolic material into the venous circulation and incomplete occlusion. In our patient, marked vessel dilatation and the inability to obtain a secure position for controlled proximal occlusion made embolization unsafe.

Akbulut et al’s^9^ review of 69 studies and 78 patients with giant splenic aneurysms identified only three concurrent AVMs, confirming their rarity, with considerable variability in management strategies and approximately 80% of patients asymptomatic at diagnosis, consistent with our case.^10^ Surgical intervention remains the mainstay of treatment, with aneurysmectomy and reconstruction favored for proximal lesions and splenectomy with *excision of the vascular* lesion for distal or hilar involvement,^9^ whereas Yadav et al^10^ similarly achieved definitive management of a large aneurysmal splenic artery and vein with an intervening AVM via complete excision and splenectomy. Treatment should be individualized according to vascular anatomy and hemodynamic characteristics. Endovascular embolization may allow splenic preservation in suitable cases; however, in high-flow shunts, coil migration into the venous circulation is a concern. When safe embolization is not feasible, splenectomy with complete excision of the vascular lesion provides definitive treatment. Portal hypertension complicates most splenic arteriovenous shunts, except in up to 16% of cases,^10^ with persistent high-pressure shunting predisposing to gastric variceal formation and potential life-threatening hemorrhage.^11^ Definitive surgical management should therefore aim to eliminate the shunt and alleviate portal hypertension.

## Conclusions

Management of splenic arteriovenous shunts should be individualized according to vascular anatomy and flow characteristics. Endovascular treatment may be considered when technically feasible, whereas splenectomy with complete shunt excision remains an effective option when embolization is unsafe.

## Funding

None.

## Disclosures

None.
